# Functional studies of *Drosophila* zinc transporters reveal the mechanism for dietary zinc absorption and regulation

**DOI:** 10.1186/1741-7007-11-101

**Published:** 2013-09-24

**Authors:** Qiuhong Qin, Xiaoxi Wang, Bing Zhou

**Affiliations:** 1 State Key Laboratory of Biomembrane and Membrane Biotechnology, School of Life Sciences, Tsinghua University, Beijing 100084, China

**Keywords:** Dietary zinc, *Drosophila*, Intracellular zinc trafficking, Regulation, Zinc absorption

## Abstract

**Background:**

Zinc is key to the function of many proteins, but the process of dietary zinc absorption is not well clarified. Current knowledge about dietary zinc absorption is fragmented, and mostly derives from incomplete mammalian studies. To gain a comprehensive picture of this process, we systematically characterized all zinc transporters (that is, the Zip and ZnT family members) for their possible roles in dietary zinc absorption in a genetically amenable model organism, *Drosophila melanogaster*.

**Results:**

A set of plasma membrane-resident zinc transporters was identified to be responsible for absorbing zinc from the lumen into the enterocyte and the subsequent exit of zinc to the circulation. dZip1 and dZip2, two functionally overlapping zinc importers, are responsible for absorbing zinc from the lumen into the enterocyte. Exit of zinc to the circulation is mediated through another two functionally overlapping zinc exporters, dZnT1, and its homolog CG5130 (dZnT77C). Somewhat surprisingly, it appears that the array of intracellular ZnT proteins, including the Golgi-resident dZnT7, is not directly involved in dietary zinc absorption. By modulating zinc status in different parts of the body, we found that regulation of dietary zinc absorption, in contrast to that of iron, is unresponsive to bodily needs or zinc status outside the gut. The zinc transporters that are involved in dietary zinc absorption, including the importers dZip1 and dZip2, and the exporter dZnT1, are respectively regulated at the RNA and protein levels by zinc in the enterocyte.

**Conclusions:**

Our study using the model organism *Drosophila* thus starts to reveal a comprehensive sketch of dietary zinc absorption and its regulatory control, a process that is still incompletely understood in mammalian organisms. The knowledge gained will act as a reference for future mammalian studies, and also enable an appreciation of this important process from an evolutionary perspective.

## Background

Zinc, an essential micronutrient, serves as a structural, catalytic, or regulatory component of many zinc-containing proteins, and is important in almost all aspects of biology [1-5]. Zinc deficiency is a worldwide health problem, and it is estimated that at least 25% of the population is at risk [6]. Low zinc can cause pleiotropic problems, such as abnormal morphogenesis, growth retardation, and dysfunction of the reproductive and immune systems [2,7-11]. Too much zinc accumulation is also harmful to cells and organisms. Maintenance of zinc homeostasis is therefore critical, and multiple mechanisms participate in ensuring the proper uptake, storage, and efflux of zinc. Zinc dyshomeostasis has been shown to cause or influence many common diseases including diabetes, neurodegeneration, and cancer [12,13].

Given the multi-faceted roles of zinc in biology and the alarmingly high deficiency rate observed in human populations, the importance of research into zinc is starting to be appreciated. Recent research indicates that zinc transport across membranes is mediated by two subfamilies of mammalian zinc transporters, ZnT (Slc30) and Zip (Slc39) [14-17]. Members of the Zip protein family function in zinc influx from the extracellular medium or vesicular organelles into the cytoplasm, and those of the ZnT family mediate zinc efflux or compartmentalization. Systemically, dietary zinc is absorbed through the enterocyte and transported into the circulation and then to the tissues where it is needed. Adjustments in zinc absorption and excretion are the primary means of maintaining zinc homeostasis [18-21].

Human *Zip4* has been identified as the gene responsible for acrodermatitis enteropathica, a disease caused by impaired absorption of dietary zinc in the intestine [22,23]. The Zip4 protein has been proposed to absorb zinc from the lumen, a role which is supported by its localization in the apical membrane of the enterocyte and its functionality in the mouse [24,25]. Expression of *Zip4* was found to be strongly responsive to dietary zinc concentrations, exhibiting upregulation with zinc limitation and downregulation with zinc excess, and thus indicating a mechanism by which the absorptive rate of dietary zinc can be beneficially regulated [22-26]. Mutations in *Zip1, Zip2, or Zip3* also confer on mice a decreased ability to survive under dietary zinc limitation, particularly during pregnancy, when zinc absorption is normally increased [27-29]. *In vitro* studies additionally showed that human Zip1 can regulate zinc homeostasis in intestinal epithelial Caco-2 cells [30]. However, direct supporting evidence for the involvement of Zip1, Zip2, and Zip3 in mammalian dietary zinc absorption is still lacking. It has been suggested that *ZnT1* on the basal membrane is involved in pumping of zinc from the cytosol of enterocytes into the circulation, and this was functionally confirmed in *Drosophila*[31].

Despite this progress, our knowledge of zinc absorption is still fragmented and limited, and mostly derived from studies in mammalian organisms, some of which were not functional studies. Targeted mutagenesis of many zinc transporters in mice have not yet been performed and for those that have been targeted, mutations were usually generated ubiquitously instead of in a tissue-specific manner, making analysis of their specific role in dietary zinc absorption difficult. For example, *Znt1* knockout mice die at the embryonic stage, precluding further functional analysis of ZnT1 in dietary zinc absorption [32]. *Znt7* mutant mice have an overall lower bodily zinc level, suggesting that *Znt7* might be a player in dietary zinc absorption, but it can be argued that this is a secondary effect due to the intracellular zinc dyshomeostasis in *Znt7*-mutant cells. Indeed, *Znt7-*mutant mice do not display typical zinc deficiency symptoms, and their phenotypes cannot be rescued by zinc supplementation [33].

As a result of these limits, after years of studies we still do not have a complete picture of dietary zinc utilization in a single platform. It remains unknown exactly how many zinc transporters are involved in dietary zinc utilization, how they are regulated, and while western and immunohistochemical evidence has shown that some intracellular zinc transporters are expressed in the mouse gastrointestinal tract [33-37], it is not known whether those transporters found on membranes of the intracellular exocytosis pathway are important for dietary zinc absorption. To gain a more comprehensive picture of zinc absorption, we took advantage of the powerful genetics of *Drosophila* and systematically dissected the specific involvement of all potential dZips and dZnTs in gut zinc absorption. Prior to this study, our understanding of dietary zinc absorption in *Drosophila* was extremely limited. Although some analyses of dZips and dZnTs have been undertaken [17,38,39], none of these transporters, except for dZnT1 [31], have been studied for their involvement or regulation in the process of dietary zinc absorption.

## Results

### Identification of two close homologs, dZip1 and dZip2, as specific zinc transporters involved in dietary zinc absorption

**Figure 1:**
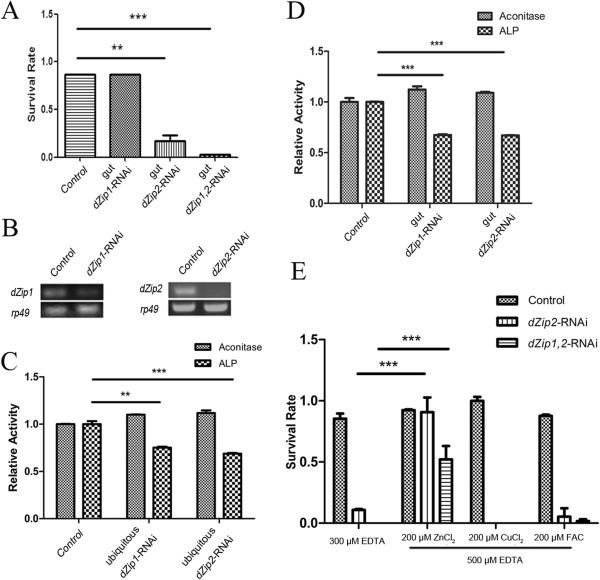
***Drosophila Zip1 *(dZip1) and dZip2 are zinc-specific transporters involved in dietary zinc absorption. (A)** Gut-specific knockdown of *dZip2* or of *dZip1 dZip2* in combination resulted in impaired larval development under zinc deficiency (0.3 mmol/l EDTA). **(B)** Reverse transcriptase (RT)-PCR analysis of the knockdown effect of the *dZip1*-RNAi and *dZip2*-RNAi lines. *rp49* was used as the loading control. **(C)** Ubiquitous knockdown of either *dZip1* or *dZip2* produced a reduction in alkaline phosphatase (ALP) activity but did not affect aconitase activity. **(D)** Gut-specific knockdown of either *dZip1* or *dZip2* similarly led to reduced ALP activity of whole body minus gut in flies fed on 0.1 mmol/l EDTA-supplemented food. **(E)***dZip2*-RNAi and *dZip1*-*, dZip2*-RNAi flies are specifically sensitive to zinc deficiency. The survival rate of these RNAi flies on EDTA-supplemented food could be rescued by adding back zinc but not other metals. **(A,D)** Genotypes of flies used were *NP3084/+*, *NP3084/+; dZip1*-RNAi/*+* (*dZip1*-RNAi fly), *NP3084/+; dZip2*-RNAi/*+* (*dZip2*-RNAi fly), *NP3084/+; dZip1-, dZip2*-RNAi/*+* (*dZip1-, dZip2*-RNAi fly). **(B,C,E)** Genotypes of flies used were *da-GAL4/+* for control and *da-GAL4/dZip1*-RNAi for *dZip1*-RNAi, *da-GAL4/dZip2*-RNAi for *dZip2*-RNAi, and *da-GAL4/dZip- dZip2*-RNAi for the double RNAi fly. Values are presented as means ± SEM; n ≥ 3. *P < 0.05, **P < 0.01, ***P < 0.001; one-way ANOVA.

We previously demonstrated that dZnT1 is involved in the efflux of zinc from the midgut enterocytes for systemic use. However, it was not known which Zip is responsible for zinc uptake into the enterocytes. According to BLASTP searches for *Drosophila* homologs of mammalian Zip family members, the *Drosophila* genome encodes 10 putative Zip proteins (see Additional file 1: Figure S1A) [17]. Notably, the *D. melanogaster* genome lacks a close homolog of Zip4, a key player in mammalian absorption of dietary zinc. This was further confirmed when hZip4 and its closest *D. melanogaster* homolog CG10006 or *foi* were used as queries to blast across all genomes of various *Drosophila* species [40], suggesting that the role of Zip4 is executed by some other Zip homologs in the fly. To identify the Zip protein that mediates zinc uptake, we knocked down individually each of these putative zinc transporters, both ubiquitously (using *daughterless-GAL4* or *da-GAL4*) and gut-specifically (using a gut-specific Gal4 line *NP3084*), and tested the sensitivity of the larvae to zinc depletion. A dramatic effect was observed with CG9430. When *CG9430* expression was knocked down, either ubiquitously or gut-specifically, only around 10 to 15% of the larvae survived to adulthood on a zinc-limited diet (0.3 mmol/l EDTA-supplemented food) whereas the eclosion of the control flies was only slightly affected under the same conditions (Figure 1A; see Additional file 1: Figure S1C), suggesting that CG9430 is indispensable for proper zinc uptake.

The protein encoded by *CG9430* shows high homology (28% identity and 50% similarity) to human Zip1 and Zip2 (hZip1 and hZip2). Interestingly, an immediately adjacent gene in this genomic region, CG9428, is very closely related to CG9430 (52% identity and 69% similarity) (see Additional file 1: Figure S1B). Compared with the CG9430 protein, the CG9428 protein displays slightly better homology to hZip1, and is in fact the closest homolog of hZip1 in the fly genome (29% identity and 48% similarity). We have therefore named CG9428 and CG9430 as *Drosophila* Zip1 (dZip1) and dZip2, respectively, hereafter. Both dZip1 and dZip2 are predicted to have the typical features of Zip family members, including eight transmembrane domains (TMDs), and extracellular amino and carboxyl termini.

The high similarity between dZip1 and dZip2 and their adjacent locations on the genome prompted us to further investigate whether dZip1 also participates in gut zinc uptake. Consistent with this notion, these two genes are the most highly expressed Zip genes in the gut, according to the fly atlas [41]. Because ubiquitous knockdown of *dZip1* did not cause significant aberrance in viability, morphology, or fertility in flies fed on either normal or EDTA food, we decided to use a more sensitive assay to measure the effect on zinc absorption.

Activity of the secretory enzyme alkaline phosphatase (ALP) is very sensitive to zinc deficiency [42-46]. Indeed, ubiquitous or gut-specific RNA interference (RNAi) of either *dZip1* or *dZip2* significantly reduced the activity of ALP, but had no effect on the activity of the iron-dependent enzyme aconitase (Figure 1C,D) [47].

When we performed RNAi of both *dZip1* and *dZip2*, and examined the additive effect when both genes were suppressed, virtually no larvae survived to adulthood, whereas a few larvae (< 10%) survived to adulthood when *dZip2* alone was knocked down (Figure 1A; see Additional file 1: Figure S1C). Adding zinc back into the EDTA-containing food was able to restore the survival rates of *dZip2*-RNAi and *dZip1, dZip2-*RNAi flies to nearly normal level and to about 50% of normal level respectively, but other metals, including copper, manganese, and iron, did not have any ameliorating effect (Figure 1E). These results suggest that dZip1 and dZip2 are zinc-specific transporters, and while both are required for dietary zinc uptake, they are functionally partially redundant as well.

### dZip1 and dZip2 are plasma membrane-resident zinc transporters responsible for zinc uptake into midgut enterocytes

During larval development, *dZip1* is mainly expressed in the midgut, and is also present in trachea and testis, according to FlyAtlas Expression Data [41]. To examine the endogenous expression pattern of dZip1 at the protein level, we raised a polyclonal antibody against dZip1, and performed immunofluorescence staining on dissected larval gut.

**Figure 2:**
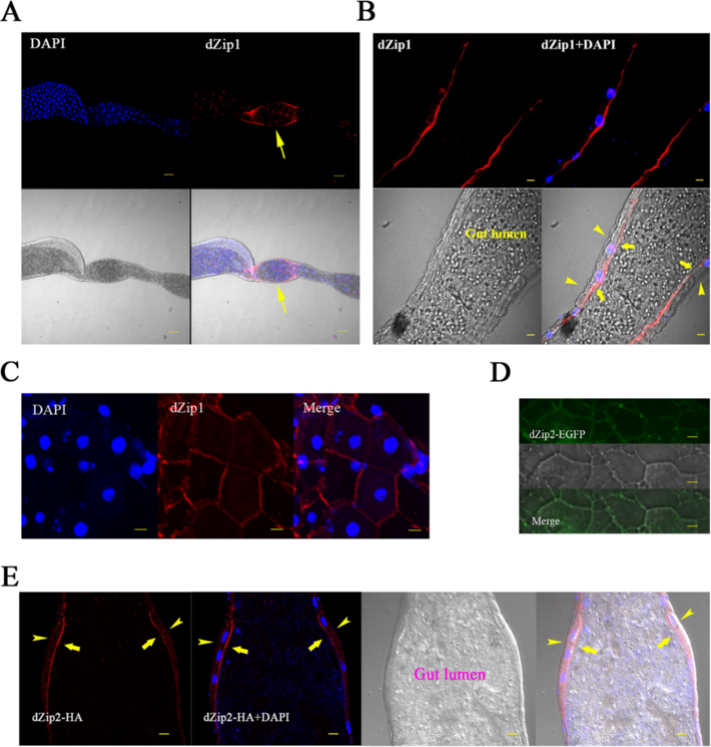
***Drosophila Zip1 *(dZip1) and dZip2 are localized to the plasma membrane. (A-C)** Specific localization of dZip1 on the apical side in the larval midgut constriction. dZip1 immunofluorescence staining is red, and nuclear DAPI staining is blue. **(A)** Specific distribution of dZip1 immunofluorescence (red) in the larval midgut constriction. Arrows denote the midgut constriction. Scale bars = 50 μm. **(B)** dZip1 (red) is located on the apical membrane of cells in the midgut constriction. Arrowheads and arrows indicate the basal and the apical membranes, respectively. Scale bars = 10 μm. **(C)** A surface view of the midgut constriction clearly shows the plasma membrane localization of dZip1 (red). Scale bars = 10 μm. **(D)** Membrane localization of dZip2 expressed in Caco-2 cells. To facilitate visualization, enhanced green fluorescent protein (eGFP)-tagged dZip2 (green) was used. Scale bars = 10 μm. **(E)** Immunofluorescence (red) against hemagglutinin (HA) reveals apical distribution of dZip2-HA in the midgut cells. DAPI (blue) shows the nucleus. Arrowheads and arrows indicate the basal and the apical membranes, respectively. Scale bars = 10 μm. Flies used are *NP3084*/*UAS-dZip2-HA.*

Intensive expression of dZip1 can be detected in the midgut constriction (Figure 2A). Under higher magnification, the endogenous dZip1 was found to be localized to the plasma membrane of the enterocytes (Figure 2C), and interestingly, it was mainly restricted to the apical membrane (Figure 2B) of the enterocytes, which lines the lumen of the midgut. This apical localization is consistent with the role of dZip1 in dietary zinc uptake.

It has been reported that dZip2, when fused to enhanced green fluorescent protein (eGFP), presents a somewhat basolateral expression in the salivary glands [38]. We therefore investigated its location, particularly in the midgut. Because there is no dZip2 antibody currently available, we fused eGFP in frame to the C terminal of dZip2, and expressed the fusion protein in human Caco-2 cells. As predicted, dZip2-eGFP was found to be located on the plasma membrane of the Caco-2 cells (Figure 2D).

To examine whether dZip2 localizes to the apical side of midgut cells, we generated a *dZip2*-HA transgenic fly by fusing an hemagglutinin (HA) tag to the C terminal of dZip2, and expressed it in the midgut. A clear signal was observed on the apical membrane of the midgut (Figure 2E). These results thus suggest that both dZip1 and dZip2 mediate the absorption of dietary zinc from lumen into the cytosol of the enterocyte.

**Figure 3:**
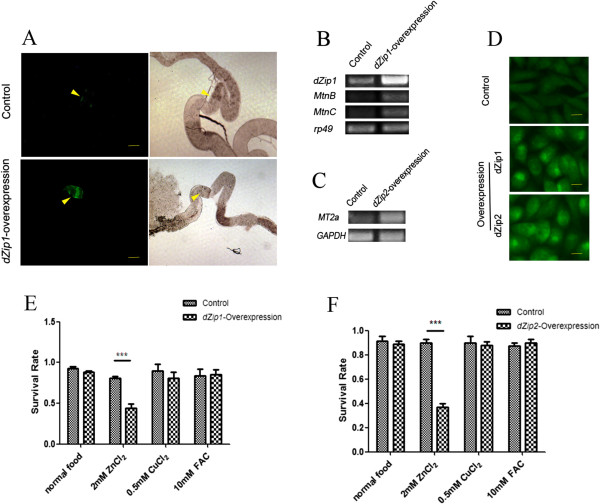
***dZip1 *or *dZip2 *overexpression causes zinc accumulation. (A)** Fluorescence of *MtnB*-eYFP was enhanced in the midgut constriction of the larvae overexpressing dZip1. Genotypes of the flies are *MtnB-EYFP/+; da-GAL4/+* for the control larvae, and *MtnB-EYFP/+; da-GAL4/UAS-dZip1* for the larvae overexpressing *dZip1*. Arrowheads denote the midgut constriction. Scale bars = 100 μm. **(B)** Reverse transcriptase (RT)-PCR analysis showing upregulation of *MtnB* and *MtnC* in larvae overexpressing *dZip1. rp49* was used as the reference gene for normalization. **(C)** RT-PCR analysis of *MT2a* transcriptional induction by overexpression of *dZip2* in Caco-2 cells. *GAPDH* was used as the reference gene for normalization. **(D)** Zinpyr-1 staining reveals intracellular zinc accumulation after dZip1 or dZip2 expression in Chinese hamster ovary (CHO) cells. Scale bars = 10 μm. **(E)** Ubiquitous overexpression of *dZip1* specifically resulted in zinc sensitivity. **(F)** Gut-specific overexpression of *dZip2* led to zinc sensitivity. Genotypes of flies are *da-GAL4/+* for the control, *da-GAL4/UAS-dZip1* for *dZip1* overexpression; *NP3084/+* for the gut-specific control fly) and *NP3084/UAS-dZip2* for the gut-specific *dZip2* overexpression fly. Values are presented as means ± SEM; n ≥ 3. *P < 0.05, **P < 0.01, ***P < 0.001; one-way ANOVA.

To confirm that dZip1 and dZip2 function as zinc importers, we monitored cytoplasmic zinc levels when they were overexpressed. We used the zinc-activating reporter *MtnB-*eYFP for this purpose. *MtnB-*eYFP comprises the regulatory sequence of the zinc-responsive gene metallothionein B (*MtnB*), an intracellular zinc binding protein, fused to an enhanced yellow fluorescent protein (eYFP) [31,48,49]. The *MtnB-*eYFP fluorescence signal was enhanced in *dZip1*-overexpressing larvae at the midgut constriction (Figure 3A), indicating excessive zinc accumulation in the cytosol of these cells. Consistent with the *MtnB-*eYFP fluorescence result, semi-quantitative reverse transcriptase (RT)-PCR also showed that both *MtnB* and *MtnC* were induced in flies with ubiquitous *dZip1* overexpression (Figure 3B) [50], and these flies displayed specific sensitivity to dietary overload of zinc, but not to dietary overload of copper or iron (Figure 3E). Zinc accumulation, as detected by the zinc indicator Zinpyr-1, was also evident when dZip1 was expressed in Chinese hamster ovary (CHO) cells (Figure 3D). These observations confirmed that overexpression of *dZip1* leads to zinc accumulation in the cytosol of cells.

Ubiquitous dZip2 overexpression leads to embryonic or first-instar larval lethality. This happened even when we used the gut-specific Gal4 driver. However, we identified a comparably weaker dZip2 line, in which ubiquitous expression still resulted in early larval lethality but gut-specific activation did not. This line, when gut-activated, exhibited zinc-specific sensitivity (Figure 3F).

We then used Caco-2 and CHO cells to study the zinc influx function of dZip2. Consistently, in *dZip2*-expressing cells, the *MT2a* transcriptional level, a reflection of cytoplasmic zinc level, was much higher than that of the control (Figure 3C). This zinc elevation was also evident when Zinpyr-1 was used as the zinc indicator (Figure 3D). These results indicate that dZip2 also transports zinc into the cytoplasm.

### Intracellular zinc transporters are not significant in dietary zinc absorption

Our aforementioned experiments, along with previously published work [31] helped us to identify dZip1 and dZip2 as being required for transport of dietary zinc into the cytoplasm of the gut cells, and dZnT1 as being required to pump zinc out of the enterocytes into the hemolymph [31]. However, these zinc transporters are all plasma membrane zinc transporters, and whether the set of intracellular zinc transporters along the secretory pathway is involved in the zinc egress process is unknown. In dietary copper absorption, for example, the Golgi-resident Menkes gene *ATP7A* is critical and patients with Menkes disease exhibit severe bodily copper shortage [51-53]. If zinc absorption resembles that of copper, we would expect certain intracellular ZnT proteins, which mediate zinc efflux into the secretory pathway to be involved. The *Drosophila* genome encodes seven putative ZnT proteins, including five possible intracellular ZnTs, as indicated by homology comparison with mammalian ZnT proteins (see Additional file 2: Figure S2A) [17].

**Figure 4:**
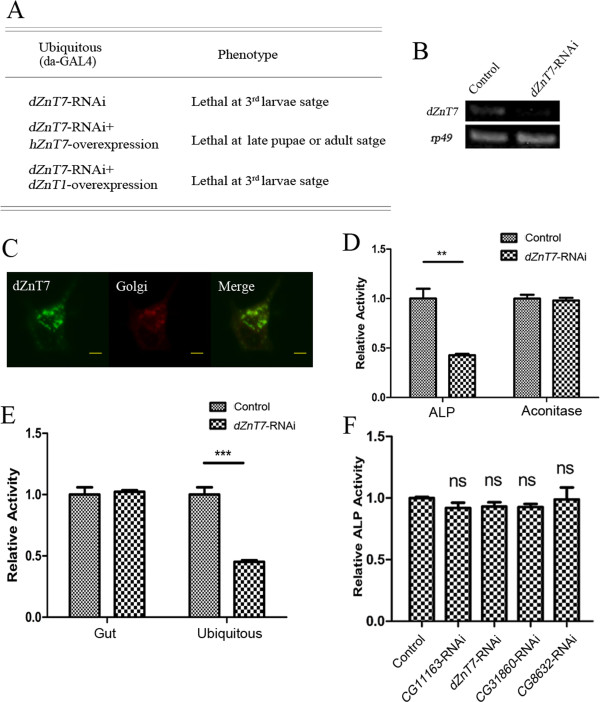
***CG6672 (dZnT7)*, the *Drosophila *ortholog of *hZnT7*, is not directly involved in dietary zinc absorption. (A)***hZnT7*, but not *dZnT1*, partially rescues *dZnT7* after RNA interference (RNAi). Ubiquitous knockdown of *dZnT7* caused lethality to the wandering larvae, but with *hZnT7* these flies reached the late pupal or even adult stage. **(B)** RT-PCR analysis of the knockdown effect of *dZnT7*-RNAi lines at third-instar larval stage. *rp49* was used as the loading control. **(C)** Golgi localization of dZnT7 in Caco-2 cells. Fluorescence markers; enhanced green fluorescent protein (eGFP)-tagged dZnT7 (green); red fluorescent protein (RFP)-tagged Golgi-targeted peptide (red); merged image (yellow) of dZnT7 and Golgi markers. Scale bars = 10 μm. **(D)** The activity of alkaline phosphatase (ALP), but not aconitase, was significantly reduced in larvae with *dZnT7*-RNAi driven by *da-GAL4*. **(E)** Gut-specific knockdown of *dZnT7* could not reduce the ALP activity of the whole body, although the ubiquitous silencing causes significant reduction. **(F)** ALP activity of whole body minus gut in gut-specific RNAi of intracellular ZnTs on zinc-limited (0.3 mmol/l EDTA) food. Neither *dZnT7*, nor any other intracellular ZnT transporters, had any discernible effect on zinc availability for the rest of the body. *NP3084* was crossed with individual RNAi lines. Values are presented as means ± SEM; n ≥ 3. *P < 0.05, **P < 0.01, ***P < 0.001; one-way ANOVA.

**Figure 5:**
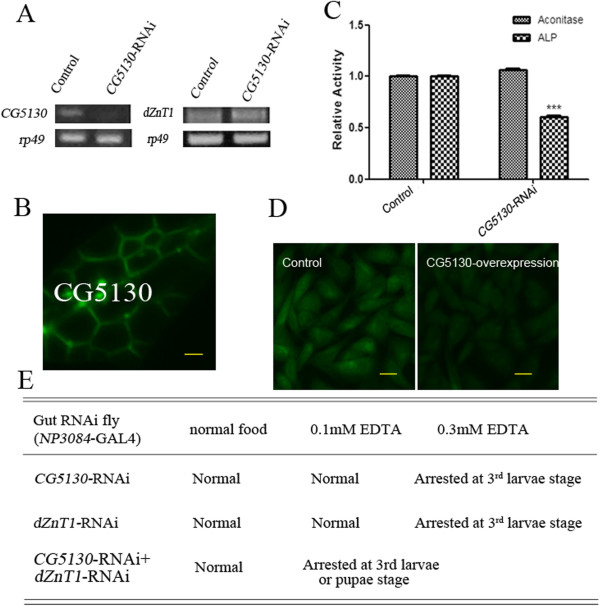
***Drosophila *ZnT1 (dZnT1) and its close homolog CG5130 export zinc from the gut for systemic use. (A)** Reverse transcriptase (RT)-PCR analysis of the knockdown effect of *CG5130*-RNAi. *rp49* was used as the loading control. **(B)** Membrane localization of CG5130 in Caco-2 cells. Enhanced green fluorescent protein (eGFP)-tagged CG5130 is shown in green. Scale bars = 10 μm. **(C)** Reduced alkaline phosphatase (ALP) activity of whole body minus gut in gut-specific *CG5130-*RNAi larvae raised on zinc-limited food (0.1 mmol/l EDTA), with little change in aconitase activity. Genotypes of the flies are *NP3084/+* (gut-specific control fly) and *NP3084/CG5130*-RNAi (gut-specific *CG5130*-RNAi fly). Values are presented as means ± SEM; n ≥ 3. *P < 0.05, **P < 0.01, ***P < 0.001; one-way ANOVA. **(D)** Zinpyr-1 staining showing intracellular zinc reduction caused by expressing CG5130 in Chinese hamster ovary (CHO) cells. Scale bars = 10 μm. **(E)** Gut-specific knockdown of *CG5130* plus *dZnT1* further exacerbated the phenotype on EDTA-supplemented food.

To analyze the functions of these ZnT proteins in gut zinc absorption, we collected all available RNAi lines from the Vienna *Drosophila* RNAi Center (VDRC), and custom-made another set at the Tsinghua Fly Center. *ZnT35C* was not further examined because it is not expressed in the midgut and was previously functionally characterized in malpighian tubules (FlyAtlas) [39]. Of the remaining ZnTs, CG31860 is not expressed in the midgut (FlyAtlas; see Additional file 2: Figure S2D); CG6672 (dZnT7) and CG5130 are analyzed in detail as shown in Figures 4 and 5; and the RNAi effect of the other two ZnTs, CG11163 and CG8632 based on RT-PCR analysis, are presented in Additional file 2 (Figure S2B,C). Most of these RNAi lines against intracellular zinc transporter genes caused lethality when ubiquitously activated (see Additional file 3: Table S1A), suggesting that these RNAi lines are working efficiently, and that the targeted ZnTs are indispensable for fly development.

However, when these intracellular zinc transporters were knocked down specifically in the gut by the gut GAL4 NP3084, none resulted in any defects in viability, morphology, or fertility, either under normal or zinc-deficient conditions (see Additional file 3: Table S1B), nor even at 29°C, a temperature at which the Gal4 protein is thought to be more potent and produces a stronger RNAi effect. To further scrutinize their involvement in dietary zinc absorption, RNAi of these intracellular transporters was carried out in a sensitized background with *dZnT1* knockdown. When *dZnT1* is knocked down, flies are more sensitive to zinc shortage (see Additional file 3: Table S1B) [31]. However, even under these conditions, the additional knock down (using gut-specific RNAi) of each of these intracellular ZnTs did not in any case noticeably increase the sensitivity of the *dZnT1*-RNAi flies to zinc deficiency (see Additional file 3: Table S1B).

As an independent and more sensitive test for bodily zinc deficiency, we quantified the ALP activity in the whole body minus the gut when these intracellular ZnTs were gut-specifically knocked down. We reasoned that a slight alteration in the zinc level might be reflected by a change in ALP, but might not result in overall survival or developmental phenotypes. We found that the ALP activity of all the gut-specific RNAi lines of intracellular ZnTs did not change (Figure 4F), indicating that intracellular zinc exocytosis is not significantly involved in dietary zinc absorption in the gut.

The Golgi apparatus is the harbor of the secretory pathway. ZnT7 has been shown to be important for the control of zinc levels in the Golgi apparatus [33-35,42,54]. A previous mouse *Znt7* knockout study presented a perplexing scenario regarding the role of ZnT7 in dietary zinc absorption: while the *Znt7*-null mouse has a low overall zinc level, its tissues are not deficient in zinc. Furthermore, zinc supplementation cannot rescue the phenotype at all [33]. If indeed exocytosis is involved in dietary zinc absorption, we would expect zinc loading by the *Drosophila* ZnT7 counterpart to play a significant role.

*Drosophila* has only one likely ZnT7 homolog, *CG6672*. To confirm that this is indeed the *Drosophila* ZnT7, first we tried to determine the subcellular location of the CG6672 protein. To facilitate visualization, we fused eGFP in frame to the C terminal of CG6672. The Golgi marker was produced by fusing red fluorescent protein (RFP) behind the Golgi-targeted peptide of human β-1,4-galactosyltransferase [55-58]. We found that when CG6672 was co-transfected into Caco-2 cells, it apparently colocalized with the Golgi marker (Figure 4C), as described in a previous report [46]. Furthermore, the larval lethality resulting from ubiquitous silencing of *CG6672* (Figure 4A) [17] could be partially rescued to late pupal or adult stage by expression of *hZnT7*, but not of *dZnT1* (Figure 4A), corroborating that CG6672, hereafter named dZnT7, is the *Drosophila* ZnT7.

ALP activity is dependent on zinc loading in the Golgi. As expected, ubiquitous knockdown of *dZnT7* significantly reduced zinc-dependent ALP activity, but had little effect on iron-dependent aconitase activity (Figure 4D). The affected flies exhibited a severe but hZnT7-rescuable phenotype: they died as late third-instar larvae. This experiment also showed the potent RNAi effect of the lines used. Consistent with the mouse knockout study, ubiquitous *dZnT7* RNAi led to an overall reduction of zinc in the whole body (Figure 4E). To address precisely the function of *dZnT7* in dietary zinc absorption, we tissue*-*specifically knocked down *dZnT7* in the gut (using *NP3084*), and then examined the effects of this knockdown on the rest of the body. *NP3084*-driven *dZnT7* RNAi did not appreciably affect the ALP activity of the whole body (Figure 4E). The above results suggest that dZnT7 in the gut does not contribute to systemic zinc levels, but rather that dZnT7 functions locally to regulate the activity of zinc-dependent enzymes. Therefore, zinc efflux to the Golgi does not seem to be significantly involved in dietary zinc absorption.

### CG5130 (dZnT77C), a close homolog of dZnT1, also participates in the exit of zinc from enterocytes for systemic use

In the process of our screen for ZnT transporters involved in zinc absorption in the gut, the knockdown of *CG5130*, localized at 77C in the genomic region, was found to cause sensitivity in flies on EDTA-supplemented food when ubiquitously or gut-specifically knocked down (Figure 5E). In the phylogenetic tree of *Drosophila* ZnTs, CG5130 lies closest to dZnT1 (see Additional file 2: Figure S2A), sharing with it 26% identity and 48% similarity. Consistent with the plasma-membrane residence of dZnT1, CG5130 is also localized to the plasma membrane as shown by the fluorescence signal emitted by CG5130-eGFP in Caco-2 cells (Figure 5B). This is consistent with the previous finding showing the basolateral membrane localization of CG5130 in salivary glands [38]. CG5130 expression in CHO cells led to zinc reduction as indicated by Zinpyr-1 staining (Figure 5D).

To further address the zinc-absorption role of CG5130 in the gut, we examined the zinc-dependent ALP activity of the rest of the body when *CG5130* was gut-specifically knocked down. Consistent with the observed sensitivity to zinc deficiency, gut-specific RNAi of *CG5130* led to decreased ALP activity of the whole body minus gut, but did not significantly change aconitase activity (Figure 5C). Considering the sequence similarity between dZnT1 and CG5130, we investigated further, and found that this RNAi effect is not mediated by reduction of dZnT1, as *CG5130* knockdown did not affect the expression of *dZnT1* (Figure 5A).

We next investigated whether dZnT1 and CG5130 function cooperatively in the exit of zinc out of the gut into the circulation. We found that when the dZnT1-RNAi line was recombined with the CG5130-RNAi line, the double RNAi line displayed greater sensitivity to zinc deficiency compared with either of the individual single RNAi lines (Figure 5E). These data indicate that zinc efflux from the enterocytes is mediated by a collaborative function of dZnT1 and CG5130.

### Expressions of the midgut zinc uptake genes are mainly influenced by changes in dietary zinc

**Figure 6:**
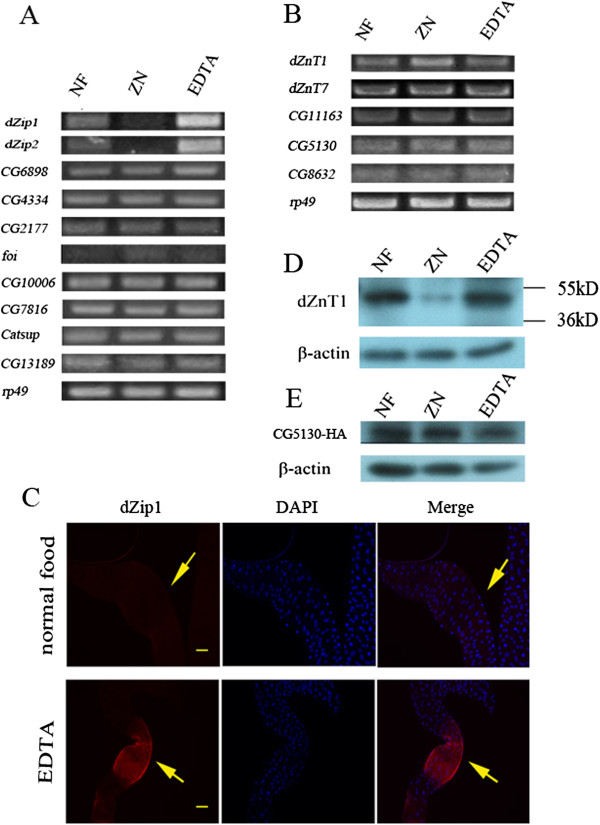
**The plasma membrane-resident *Drosophila *zinc transporters, dZips and dZnTs, are regulated by zinc availability. (A-B)** Reverse transcriptase (RT)-PCR analysis of **(A)** dZips and **(B)** dZnTs in the gut showing transcriptional responsiveness of zinc transporters to dietary zinc. **(C)** Immunostaining reveals that dZip1 is upregulated by dietary zinc limitation at the midgut constriction (arrows ). Scale bars = 50 μm. **(D,E)** Western blotting analysis shows that **(D)** dZnT1 is downregulated by dietary zinc overload, but not by zinc limitation; and **(E)** using anti-HA antibody, shows that CG5130-HA is not responsive to dietary zinc level. NF, normal food; ZN, 2 mmol/l ZnCl_2_; EDTA, 0.3 mmol/l EDTA.

One important question in the regulation of zinc absorption is how zinc transporters are regulated by available zinc. From the physiological point of view, the expression of zinc uptake proteins dZip1 and dZip2 should ideally be reduced when the diet is rich in zinc to avoid excessive zinc toxicity, and their expression should be increased when the diet is low in zinc, in order to facilitate dietary zinc uptake from zinc-limited food. Using semi-quantitative RT-PCR analysis (Figure 6), we found that this is indeed the case. Of all the *Drosophila* Zip genes, only *dZip1* and *dZip2* appear to be transcriptionally regulated by dietary zinc levels (Figure 6A). When the food was replete with zinc the RNA levels of these two zinc uptake proteins, dZip1 and dZip2, were repressed, and conversely, when the food was depleted in zinc, the RNA levels of dZip1 and dZip2 were increased. The induced dZip1 expression was further confirmed by immunostaining at the midgut constriction when dietary zinc was limited (Figure 6C).

These data suggest that dZip1 and dZip2 respond to dietary zinc availability, and function cooperatively to ensure appropriate zinc absorption under zinc-limited and zinc-supplemented conditions.

Assessing the transcription of all the ZnT genes in response to excessive dietary zinc, we detected a slight increase in expression only in the case of *dZnT1* (Figure 6B). The immunohistochemical staining results suggest that most of this increase can be explained by *de novo* or ectopic induction of ZnT1 expression in regions other than the midgut constriction region [31]. This lack of obvious transcriptional control in the zinc absorption area (the midgut constriction region) prompted us to investigate whether dZnT1 is subject to post-transcriptional control. Quantitative analysis of dZnT1 in the midgut by western blotting demonstrated a strong post-transcriptional regulation of dZnT1; when zinc was high, the dZnT1 protein was dramatically reduced (Figure 6D).

We also assayed the expression of CG5130 by fusing it to an HA tag at the C terminal. However, there was no obvious change in the CG5130 protein level in response to zinc fluctuations (Figure 6E).

### The midgut zinc uptake genes are unresponsive to bodily zinc status

**Figure 7:**
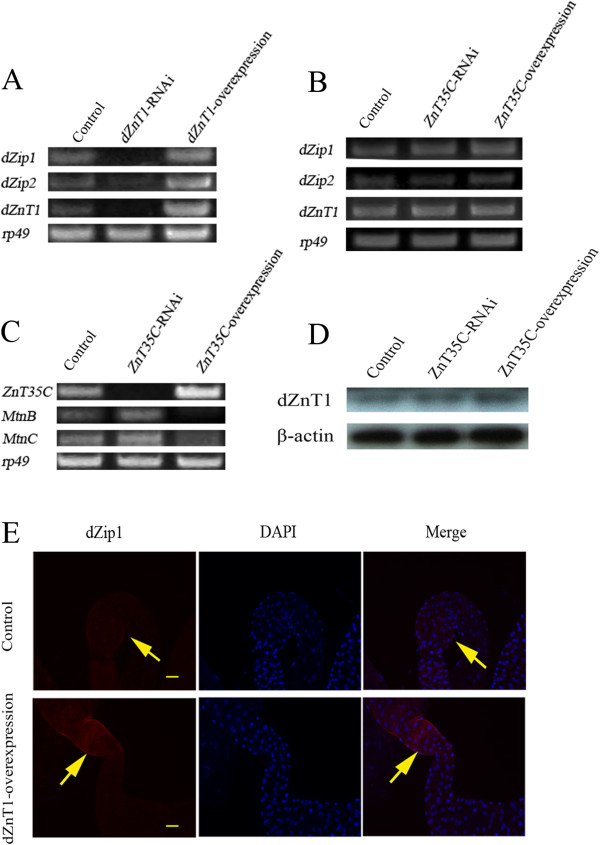
**Dietary zinc absorption is influenced by zinc levels in the enterocyte but not the rest of the body. (A)** Transcriptional responses of *dZip1* and *dZip2* to gut-specific zinc changes as revealed by reverse transcriptase (RT)-PCR analysis. **(B)** Transcription of *dZip1* and *dZip2* in the gut does not respond noticeably to body zinc changes achieved by modulating the zinc excretion process (*ZnT35C* knockdown or *ZnT35C* overexpression in the malpighian tubules). **(C)** As a control, *ZnT35C* knockdown in the malpighian tubules effectively induced the bodily transcription of *MtnB* and *MtnC* (reflecting zinc increase), whereas overexpression of *ZnT35C* repressed the transcription of *MtnB* and *MtnC*. **(D)** Western blotting analysis shows that dZnT1 expression in the enterocyte is not altered when zinc excretion is modulated. **(E)** Immunofluorescence staining shows that dZip1 is induced at the midgut constriction (arrows) when dZnT1 is specifically overexpressed in the gut, in the absence of dietary zinc change. Scale bars = 50 μm. Genotypes of flies are *NP3084*/+ (control fly) and *NP3084*/*dZnT1*-RNAi (*dZnT1*-RNAi fly), *NP3084*/+; *UAS-dZnT1*/+ (*dZnT1* overexpression fly), *NP1093*/+ (control fly), *NP1093*/+; *ZnT35C*-RNAi/+ (*ZnT35C* RNAi fly), and *NP1093*/+; *UAS-ZnT35C* (*ZnT35C* overexpression fly). RNAi, RNA interference.

Limiting zinc efflux when zinc is replete will benefit the rest of the body at the expense of the gut. This led us to investigate another important zinc regulation question: does zinc absorption reflect the bodily zinc requirement? To address this question, we genetically manipulated *Drosophila* to create these scenarios: high zinc in the rest of the body but low in the gut; high zinc in the gut but low in the body. We then determined how the influx and efflux zinc transporters responded. By introducing *dZnT1* RNAi, we could make the fly zinc replete in the gut but zinc deficient in the rest of the body [31]. Under this scenario, dZip1 and dZip2 expression was much reduced (Figure 7A), despite the unsatisfied need for zinc in the body. By overexpressing *dZnT1*, we created the opposite scenario: higher zinc level in the body but lower in the gut [31]. In this case, *dZip1* and *dZip2* were both up-regulated (Figure 7A). Using dZip1 antibody, we can clearly see a much stronger signal of dZip1 when dZnT1 is over-expressed (Figure 7E). Another way to control body zinc level is through manipulating *ZnT35C* expression [39]. When ZnT35C is repressed or over-expressed, the body zinc level is correspondingly increased or decreased (Figure 7C) [39]. Again, we did not see any alteration of dZip1 and dZip2 expression (Figure 7B). Further, we did not see obvious dZnT1 expression change either (Figure 7D).

Taking these results together, we conclude that dietary zinc uptake is not responsive to the zinc status or need of the body. The regulation is, strictly speaking, controlled by the zinc status of the enterocytes, and not even directly by the diet itself. Dietary zinc influenced the expression of these uptake genes by affecting the zinc levels of the enterocytes.

## Discussion

**Figure 8:**
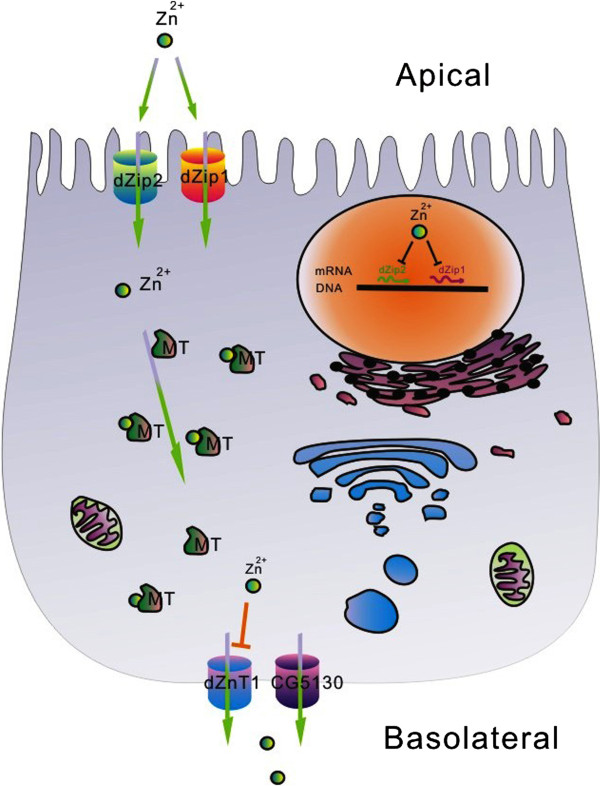
**A model for dietary zinc absorption in *Drosophila *enterocytes.** Dietary zinc absorption takes a direct transversing path through the midgut cells, bypassing the intracellular organelles. Zinc uptake from the lumen involves the *Drosophila* transporters dZip1 and dZip2 on the apical membrane, and these transporters are negatively affected at the RNA level by zinc in the enterocytes. Cytosolic zinc is pumped out into circulation by dZnT1 and CG5130 on the basolateral membrane. dZnT1 is post-transcriptionally repressed by zinc overload, whereas CG5130 is unresponsive to dietary zinc changes. Zinc in the cytosol is bound by metallothioneins to prevent its toxicity when overloaded. Zinc transporters along the secretion pathway are not directly involved in zinc efflux to the circulation. The regulated expression the zinc transporters mediating dietary zinc uptake depends autonomously on the zinc level in the enterocyte *per se*, and is unresponsive to the zinc status in the rest of the body. The negative regulation of zinc transporters by dietary zinc occurs through altering the zinc levels in the enterocyte.

In this work, we systematically dissected the repertoire of zinc transporter candidates for their functions in dietary zinc absorption. Through this systematic work in a *Drosophila* model, the mechanism of zinc absorption has started to take shape, and the important players have been clarified. Zinc absorption is mediated by several transporters localized to the plasma membrane, whereas transporters in the exocytosis pathway appear to be insignificant in this process. The regulation of dietary zinc absorption is mediated through changes in the expression of some of these plasma membrane-localized transporters. A model summarizing this process is shown in Figure 8.

In order to analyze the tissue-specific functions of these zinc transporters, we utilized tissue-specific RNAi. One caveat of this approach is that when a phenotype is seen, it is not clear whether this is due to an off-target effect, and when no effect is observed, it is not clear whether this is due to low RNAi efficiency.

To address the first concern, we used multiple RNAi lines that were generated by targeting different gene regions, and we also tested their zinc-specific effects. For example, if an RNAi line has a zinc-responsive phenotype, this suggests that the resultant phenotype is probably real, and unlikely to be the result of off-targeting because the probability of a random off-targeting event happening to a zinc metabolism gene is low.

The second concern is more of an issue because RNAi is never a genetic null, so to argue that a gene is not involved in a process simply because no phenotype is associated with RNAi needs extra caution. For the purpose of this study, we wanted to test whether intracellular ZnTs are involved in dietary zinc absorption. Gut-specific RNAi of the plasma membrane-resident ZnT genes *dZnT1* and *CG5130* resulted in a change in the zinc phenotype of the body, while gut-specific RNAi of the intracellular ZnTs had no discernible effect on dietary zinc absorption. When we performed a ubiquitous knockdown we saw specific effects for many of these intracellular ZnT genes, suggesting effective RNAi and their functionality in other aspects of zinc metabolism.

Ubiquitous RNAi of two of these genes in particular, the Golgi-resident dZnT7 and the vesicle-resident CG31860 (dZnT4 homolog), induced larval or embryonic lethality in treated flies (see Additional file 3: Table S1A) [17], however, specifically targeting each of the intracellular transporters by RNAi in the gut did not appear to have any effect on dietary zinc absorption. Worth mentioning is that CG31860 is the closest *Drosophila* homolog of CDF-2, which has been shown to function in zinc storage in the gut granules of *Caenorhabditis elegans*. Further, in *Drosophila*, suppression of *CG31860* greatly decreases MtnB-eYFP expression [17]. It appears therefore that CG31860 is an important regulator of intracellular zinc homeostasis, although it plays an insignificant role in the process of dietary zinc absorption.

The exocytosis pathway involves many organelles. In contrast to the dramatic effect seen for the plasma membrane-resident ZnTs, none of the multiple RNAi lines for the whole set of ZnTs located in different parts of this pathway displayed any zinc absorption defect, and this was the case even in an sensitized background (*dZnT1* RNAi) and using the sensitive ALP activity assay. This strongly suggests that the intracellular pathway plays no role or at least no significant role in dietary zinc absorption.

A central part of the secretion pathway is the Golgi apparatus. There is only one Golgi-resident ZnT member, dZnT7, in the fly. ZnT7 has been previously implicated in dietary zinc absorption in a mouse knock-out study [33], and *Znt7*-null mice have a reduced body zinc level. Nevertheless their phenotype is not ameliorated by intravascular zinc injection. We consider the complex phenotype of *Znt7*-null mice as arising from a universal Golgi zinc transport defect, resulting lack of zinc in the Golgi apparatus, instead of a systemic zinc deficiency. In our study, we found that universal knockdown of *dZnT7* also caused a severe phenotype, including a reduction in bodily zinc level. However, gut-specific *dZnT7* knockdown produced little effect on bodily zinc metabolism, except for a local change of the Golgi zinc level, as reflected by ALP activity reduction in the gut only.

Among the several zinc transporters involved in dietary zinc absorption, the two Zips (dZip1 and dZip2) and at least one dZnT (dZnT1) involved in zinc uptake are strongly influenced by the dietary zinc level. When there is an excess of zinc available, uptake and efflux of zinc are reduced. When zinc is scarce, the uptake transporters dZip1 and dZip2 are upregulated, whereas the efflux of zinc is not greatly affected. The ectopic induction of dZnT1 in otherwise non-expressing gut tissues may be a self-protecting mechanism for the gut when zinc overdosing happens, because zinc accumulation is generally toxic to cells.

Some general characterization of the Zip and ZnT transporters was previously performed [17,38], although the roles of different zinc transporters in the process of dietary zinc absorption were not previously explored except that of dZnT1. In this study, the zinc transporters that were newly found to be involved in dietary zinc absorption were characterized in more detail at the molecular and cellular levels. Our results, when applicable, are largely consistent with previous work, but some inconsistencies do arise. It was previously reported that ubiquitous dZip2 knockdown resulted in lethality [17]We did not see this lethality under normal culturing conditions. However, we did see lethality when the zinc supply was lowered. The difference in results is likely attributable to different culturing conditions in different laboratories such as variations in food mineral content. Likewise, midgut-specific overexpression of dZip1 was reported to be lethal [17], but in our hands this happened only when extra zinc was added. Further, we found that ubiquitous knockdown of *dZip1* produced a reduced ALP phenotype without much of a decrease in viability, whereas Lye *et al*. [17] found that ubiquitous knockdown of *dZip1* with a stronger Gal4 driver, tubulin-Gal4, resulted in larval lethality. This discrepancy may be a consequence of either the difference in the drivers used, or in the food nutrient supply, as mentioned above. Therefore, in the characterization of the general properties of these zinc transporters, our results are largely consistent with previous studies, except in a few cases in which the extent of the phenotype is different, which could be attributable to differences in the experimental conditions used.

Partial redundancy of the influx and efflux transporters were seen in dietary zinc absorption in *Drosophila*. dZip1 and dZip2 are responsible for zinc uptake, whereas dZnT1 and CG5130 are responsible for the efflux. Knocking any of these transporters down caused zinc deficiency under zinc-limited conditions, suggesting that all of them are important in the absorption of dietary zinc.

Nevertheless, slight differences do exist. Although downregulation of either dZip1 or dZip2, had equivalent effects on ALP levels when flies were cultured on 0.1 mmol/l EDTA food, on 0.3 mM EDTA food dZip1 knockdown flies developed well while *dZip2* flies were arrested at the early larval stage (Figure 1A). It is possible that dZip2 is slightly more important than dZip1 or is a more potent zinc transporter when zinc is severely depleted. This notion is consistent with the observation that overexpression of dZip2 causes a more severe phenotype than does overexpression of dZip1.

In the case of the zinc efflux transporters, dZnT1 and CG5130, knockdown of either presents a similar phenotype, namely, death at around the third-instar larval stage when zinc is limited. Interestingly, dZnT1 is regulated by zinc, whereas CG5130 is not, suggesting that dZnT1 is a regulatory transporter whereas CG5130 may play a more constitutive role in zinc absorption in the gut. Further, overexpression of dZnT1 produced a zinc-sensitive phenotype whereas CG5130 overexpression did not. We speculate that CG5130 might have a lower zinc-transporting capacity so that under conditions of zinc excess, the amount of zinc it can transport is limited, so that zinc toxicity is avoided. Alternatively, CG5130 might work together with dZnT1 (for example by forming a complex) to export zinc. Either scenario can explain why it is not important for CG5130 to have zinc-responsive expression.

Together our results offer a glimpse into the overall process of dietary zinc absorption in a *Drosophila* model. Zinc absorption, regulated by uptake, occurs through a relatively direct route from the gut lumen to the basolateral sides of the gut epithelium and hence into the circulation. Although most intracellular ZnTs are expressed in the gastrointestinal tract, these intracellular zinc transporters are not significant in dietary zinc absorption. dZip1/2 and dZnT1/CG5130 are respectively responsible for zinc intake and efflux in the enterocytes. In human studies, the *dZip1/2* counterpart *hZip4* was shown to be important for zinc absorption. *hZip4* is haploinsufficient as mutation in one allele may cause zinc deficiency [24]. Whether any other Zips are involved in this process in mammals is not well-established [30]. The lack of an ortholog of Zip4 in *Drosophila* makes it possible that dZip1/2 may substitute for Zip4 in mediating zinc absorption. Therefore, the specific transporters for a particular role may vary between different organisms. For zinc efflux, participation of mammalian *Znt1*, the counterpart of *dZnT1*, is almost a certainty, but whether *Znt1* is the sole player in zinc efflux in the mammalian gut remains to be seen. Overall, our results are notably consistent with some findings regarding mammalian zinc absorption: expression of Zip4 and ZnT1 is reduced when the diet is replete in zinc [59], and the zinc status of the body appears to have little effect on the efficiency of dietary zinc absorption [60].

## Conclusions

The genetic amenability of *Drosophila* enabled us to show which zinc transporters are involved in the process of dietary zinc absorption, and which are not. We found that dietary zinc absorption is mediated by a set of plasma membrane-resident zinc transporters with partially overlapping functions, including the importers dZip1/dZip2 and the exporters dZnT1/CG5130 (dZnT77C). The array of intracellular zinc transporters, such as the Golgi-resident ZnT7, is not involved in dietary zinc absorption. Zinc absorption is subject at the influx side to the RNA-level control of zinc importers, and at the efflux side to the translational or post-translational control of the exporters. Bodily zinc needs do not exert a feedback control on zinc absorption; instead, zinc absorption is controlled solely by the zinc levels in the enterocytes. Dietary zinc influences zinc absorption through its effects on enterocyte zinc. This work assessed for the first time all potential zinc transporters for their roles in dietary zinc absorption, and we have outlined a relatively intact picture of the dietary zinc absorption and its control in a model organism (Figure 8). The deciphering of dietary zinc absorption in *Drosophila* should be helpful for zinc absorption research in general, and offers a reference point for future studies in other organisms including comparative evolutionary analysis of this important biological process.

## Methods

### Plasmids

UAS-*dZip1* and UAS-*dZip2* were generated by PCR amplification of the coding region of *dZip1* and *dZip2*, respectively, from *Drosophila* cDNA, and cloned into vector pUAST [50]. Following this, pCDNA3.1-*dZip2-eGFP*, pCDNA3.1-*CG6672-eGFP* and pCDNA3.1-*CG5130-eGFP* were generated by fusing the enhanced green fluorescent protein (eGFP) in frame to the C terminal of the individual coding region of dZip2, C6672, and CG5130, respectively. All the constructs were verified by sequencing.

### Cell culture and transient transfection

Human Caco-2 cells were maintained in DMEM medium (Invitrogen Corp., Carlsbad, CA, USA) containing 10% fetal bovine serum (FBS; Gibco BRL, Gaithersburg, MD, USA) at 37°C. Caco-2 cells were cultured and when they reached 80% confluence, they were transfected using Lipofectamine™ 2000 (Invitrogen) in accordance with the manufacturer’s instructions. A DNA: liposome ratio of 1.6 μg: 4 μl was used for each 4 cm^2^ dish (0.8 μg DNA each when two plasmids were co-transfected).

### Fly stocks, culture media, and transgenics

Fly stocks were raised at 18°C, and all the experiments were performed at 25°C on standard cornmeal food. When necessary, the food was supplemented with EDTA, or ZnCl_2_ at the concentrations stated below for each experiment. The temperature was increased to 29°C when necessary. Fly stocks, including *da-GAL4* and UAS-*eGFP*, were obtained from Bloomington Stock Center (Bloomington, IN, USA), while *NP3084* and *NP1093* lines were obtained from the *Drosophila* Genetic Resource Center at the Kyoto Institute of Technology (Kyoto, Japan). The RNAi lines were provided by the VDRC (Vienna, Austria) or custom-made at Tsinghua Fly Center (Beijing, China). Transgenic flies were prepared by P-element-mediated transformation in *w*^*1118*^ background.

### Fly survival assays

*da-GAL4* or *NP3084* homozygous flies were crossed with transgenic flies, as indicated in each experiment. The progeny were fed on food supplemented with the stated metals or metal chelators. The density of each vial was about 70 progeny.

### Antiserum preparation

A rabbit polyclonal anti-dZip1 antibody was raised against a synthetic peptide of amino acids 173 to 186 of dZip1 (DTEPQPHKDHHGHS) by Abmart (Shanghai, China).

### Western blotting, immunohistochemistry, and microscopy

For western blot analysis, midgut samples were dissected, and homogenized in buffer containing 1% TritonX-100 in the presence of a proteinase inhibitor cocktail (P2714-1BTL’ Sigma Chemical Co., Chicago, IL, USA), centrifuged, separated by SDS-PAGE in 12% gels, and transferred to PVDF membranes (Millipore Corp., Billerica, MA, USA).

The antibodies used were anti-dZnT1, mouse monoclonal anti-β-actin, anti-HA, HRP-conjugated goat anti-mouse IgG, and HRP-conjugated goat anti-rabbit IgG (Zhongshan Goldenbridge Biotechnology, Beijing, China). Signals were developed with a SuperSignal West Dura kit (Thermo, Rockford, IL, USA). For immunohistochemistry, the anti-dZip1 antibody was preabsorbed with fixed *w*^*1118*^ embryos before being used for staining fly tissues. Third-instar larvae were dissected in cold phosphate-buffered saline (PBS), fixed with paraformaldehyde, and stained as previously described [31]. Anti-dZip1 and anti-HA were used (1:200 dilution) in combination with TRITC-conjugated goat anti-rabbit IgG (Zhongshan Goldenbridge Biotechnology Co. Ltd., Beijing, China). Samples were incubated in 50 ng/ml DAPI for 10 min for nuclear staining. Tissues were mounted in 50% glycerol/50%PBS. The fluorescence of *MtnB-*eYFP in the larval gut was captured with an ECLIPSE 80i microscope attached to a DXM1200F digital camera (both Nikon, Tokyo, Japan) The fluorescence of cell cultures was recorded by fluorescence microscopy (1X71; Olympus, Tokyo, Japan), and the immunostainings were examined using a laser scanning confocal microscope (FV500; Olympus, Tokyo, Japan).

### Zinpyr-1 staining for intracellular labile Zn^2+^

dZip1, dZip2, and CG5130 were cloned into the pIRESneo vector, and transfected into CHO cells. Stable lines were obtained by selecting with 800 μg/ml G418. Cells were incubated in DMEM containing 75 μmol/l ZnCl_2_ for 3 hours, washed 3 times with PBS for 5 minutes each time, and then fixed with paraformaldehyde for 30 minutes. After three washes, cells were incubated in 2 μmol/l Zinpyr-1 (Santa Cruz Biotechnology, Santa Cruz, CA, USA) in PBS for 30 minutes at 37°C. Excessive Zinpyr-1 was washed away by three washes in PBS, and the fluorescence signal was examined under an Olympus 1X71 fluorescence microscope.

### RNA isolation and semi-quantitative RT-PCR

Total RNA was extracted from embryos, larvae, or gut from the third-instar larvae or from cultured cells, using TRIzol reagent (Invitrogen, Carlsbad, CA, USA). cDNA was transcribed from 1 μg total RNA with TransScript First-Strand cDNA Synthesis SuperMix (TransGen Biotech Co., Ltd., Beijing, China), in accordance with the manufacturer’s instructions. Semi-quantitative RT-PCR was performed using the specific primers corresponding to partial regions of the analyzed genes.

### Measurement of ALP activity

Total protein (1 to 10 μg) was lysed in ALP lysis buffer (1.0 mmol/l Tris–HCl pH7.4, 0.5 mmol/l MgCl_2_, and 0.1% Triton X-100), then 90 μl solution A (1.0 mol/l diethanolamine, 0.5 mmol/l MgCl_2_ pH9.8) and 10 μl solution B (150 mmol/l p-nitrophenyl phosphate) were added. The absorbance at 405 nm was measured after incubation for 30 min at 25°C.

### Measurement of aconitase activity

Total protein (10 to 100 μg) was incubated in PBS with Triton (137 mmol/l NaCl, 2.7 mmol/l KCl, 10 mmol/l Na_2_HPO_4_, 2 mmol/l KH_2_PO_4_, and 0.1% Triton X-100), and then mixed with 700 μl citrate reaction solution (30 mmol/l citric acid, 50 mmol/l K_2_HPO_4_ pH 7.4). The absorbance of the reactant at 240 nm was recorded for 2 min using a UV–Visible spectrophotometer, and the aconitase activity was calculated by the increased value of the absorbance.

### Statistical analysis

Differences were analyzed by one-way ANOVA using SPSS software (version 16.0 for Windows; SPSS Inc., an IBM Company, Chicago, IL, USA). *P* < 0.05 was considered significant.

## Competing interests

The authors declare that they have no competing interests.

## Authors’ contributions

BZ conceived of the experiments, and QQ and XW performed the experiments. All authors contributed to the data analysis and manuscript writing. All authors have read and approved the final manuscript.

## Supplementary Material

Additional file 1: Figure S1 *Drosophila dZip1* and *dZip2* are two adjacent Zip family members involved in zinc uptake. **(A)** Phylogenetic tree revealing the relationship between human and *Drosophila* Zip members. All human Zips were used individually as queries in a series of BLASTP searches in the genome of *D. melanogaster*. The tree was generated by using ClustalX (version 1.81) and displayed in Treeview. This tree analysis result is consistent with that of a previous report.^17^**(B)** Relative genomic location of *dZip1* (*CG9428*) and *dZip2* (*CG9430*). Both genes are located at postion 42C6 of the chromosome 2 in *Drosophila melanogaster*, suggesting a recent evolutionary duplication. **(C)** Under conditions of zinc deficiency (0.3 mmol/I EDTA), there was impaired development of larvae after ubiquitous knockdown by RNA interference (RNAi): *dZip2* RNAi and *dZip1 dZip2* double RNAi (*dZip1, dZip2* RNAi). The genotypes of flies are *da-GAL4/+* for the control and *da-GAL4/dZip1*-RNAi for *dZip1*-RNAi, *da-GAL4/dZip2*-RNAi for *dZip2*-RNAi, and *da-GAL4/dZip1-, dZip2*-RNAi for the double RNAi fly. Data are presented as means ± SEM; n≥3. *P<0.05, **P<0.01, ***P<0.001; one-way ANOVA. Click here for file

Additional file 2: Figure S2 Phylogenetic analysis of ZnTs in *Drosophila* (compared with those in human) and the RNA interference (RNAi) effects of some dZnT lines. RNAi lines were obtained from the Vienna *Drosophila* RNAi Center (VDRC) or custom-made in the Tsinghua Fly Center. **(A)** Phylogenetic tree revealing the relationship between human and *Drosophila* ZnT members. The predicted intracellular ZnTs are in pink. All human ZnTs were used individually as queries in a series of BLASTP searches of the genome of *Drosophila melanogaster*. The tree was generated by using ClustalX (version 1.81) and displayed n Treeview. **(B-D)** Reverse transcriptase (RT)-PCR analysis of the gut-specific knockdown effect of RNAi lines of **(B)***CG11163* and **(C)***CG8632*. **(D)** Expression of *CG31860* was not detected in the gut. *rp49* was used as the loading control. Analysis of *CG6672* (*dZnT7*) and *CG5130* (*dZnT1h)* are described in Figures 4 and 5 respectively. Click here for file

Additional file 3: Table S1 Analysis of intracellular ZnTs for their roles in dietary zinc absorption in *Drosophila*. **(A)** Phenotypes of individual RNA interference (RNAi) lines driven by *da-GAL4*, a ubiquitous GAL4 line, under normal or zinc-limited conditions. **(B)** Phenotypic analysis of midgut-specific knockdown of individual RNAi lines on normal or zinc-limited food. *NP3084* was used as the midgut GAL4 driver. Click here for file
