# Supplementary material for: Functional studies of Drosophila zinc transporters reveal the mechanism for dietary zinc absorption and regulation
Source: BMC Biol. 2013 Sep 24;11:101. doi: 10.1186/1741-7007-11-101 (PMC4015762; doi:10.1186/1741-7007-11-101)
Supplement: Additional file 1: Figure S1 — Drosophila dZip1 and dZip2 are two adjacent Zip family members involved in zinc uptake. (A) Phylogenetic tree revealing the relationship between human and Drosophila Zip members. All human Zips were used individually as queries in a series of BLASTP searches in the genome of D. melanogaster. The tree was generated by using ClustalX (version 1.81) and displayed in Treeview. This tree analysis result is consistent with that of a previous report.17(B) Relative genomic location of dZip1 (CG9428) and dZip2 (CG9430). Both genes are located at postion 42C6 of the chromosome 2 in Drosophila melanogaster, suggesting a recent evolutionary duplication. (C) Under conditions of zinc deficiency (0.3 mmol/I EDTA), there was impaired development of larvae after ubiquitous knockdown by RNA interference (RNAi): dZip2 RNAi and dZip1 dZip2 double RNAi (dZip1, dZip2 RNAi). The genotypes of flies are da-GAL4/+ for the control and da-GAL4/dZip1-RNAi for dZip1-RNAi, da-GAL4/dZip2-RNAi for dZip2-RNAi, and da-GAL4/dZip1-, dZip2-RNAi for the double RNAi fly. Data are presented as means ± SEM; n≥3. *P<0.05, **P<0.01, ***P<0.001; one-way ANOVA. [file 1741-7007-11-101-S1.doc]

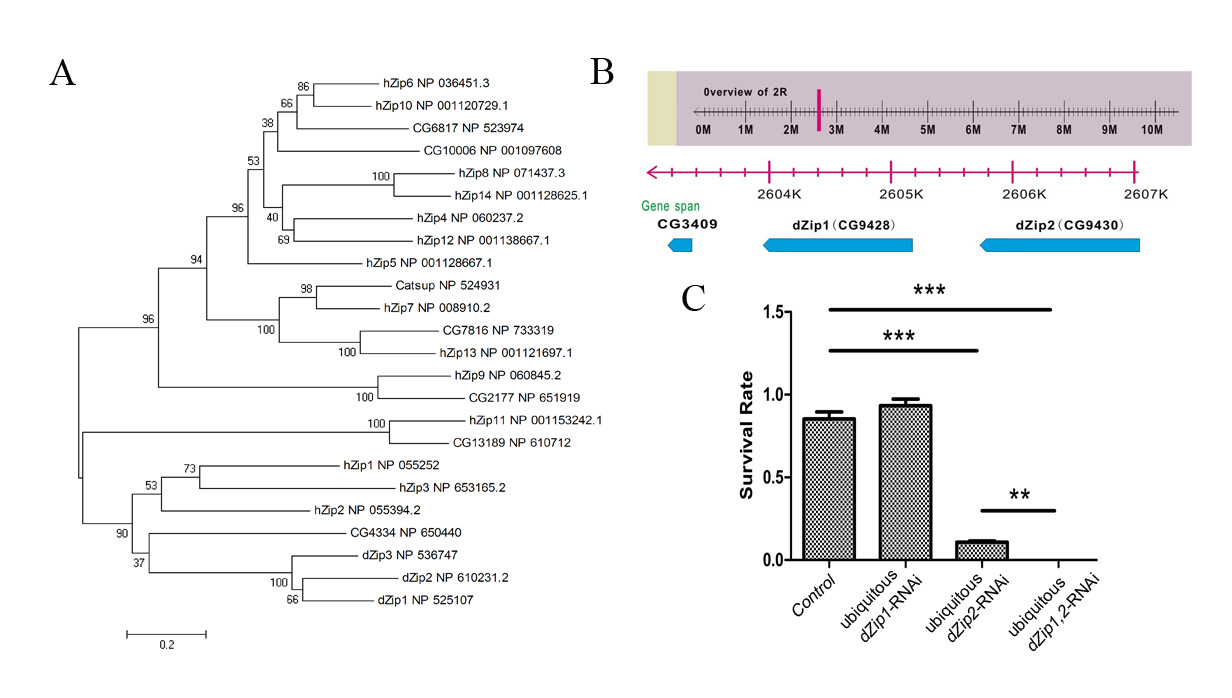


**Figure S1**. *Drosophila* *dZip1* and *dZip2* are two adjacent Zip family members involved in zinc uptake. A) Phylogenetic tree revealing the relationship among human and *Drosophila* Zip members. All human Zips were used respectively as queries to do series of BLASTP searches in the genome of *D. melanogaster*. Tree was generated by using ClustalX version 1.81 and displayed by Treeview. This tree analysis result is consistent with that of a previous report (17). B) Relative genomic location of *dZip1* (*CG9428*) and *dZip2* (*CG9430*). Both genes are at 42C6 of the 2^nd^ chromosome in *Drosophila melanogaster*, suggesting a recent evolutionary duplication. C) Impaired development of *dZip2*-RNAi and *dZip1, dZip2* double RNAi (*dZip1-, dZip2*-RNAi) larvae under zinc deficiency (0.3mM EDTA), when ubiquitously knocked-down. Genotypes of flies are *da-GAL4/+* for the control and *da-GAL4/dZip1*-RNAi for *dZip1*-RNAi, *da-GAL4/dZip2*-RNAi for *dZip2*-RNAi, and *da-GAL4/dZip1-, dZip2*-RNAi for the double RNAi fly. Data are presented as means±SEM.; n≥3. *P<0.05, **P<0.01, ***P<0.001; one-way ANOVA.
