# Supplementary material for: Functional studies of Drosophila zinc transporters reveal the mechanism for dietary zinc absorption and regulation
Source: BMC Biol. 2013 Sep 24;11:101. doi: 10.1186/1741-7007-11-101 (PMC4015762; doi:10.1186/1741-7007-11-101)
Supplement: Additional file 2: Figure S2 — Phylogenetic analysis of ZnTs in Drosophila (compared with those in human) and the RNA interference (RNAi) effects of some dZnT lines. RNAi lines were obtained from the Vienna Drosophila RNAi Center (VDRC) or custom-made in the Tsinghua Fly Center. (A) Phylogenetic tree revealing the relationship between human and Drosophila ZnT members. The predicted intracellular ZnTs are in pink. All human ZnTs were used individually as queries in a series of BLASTP searches of the genome of Drosophila melanogaster. The tree was generated by using ClustalX (version 1.81) and displayed n Treeview. (B-D) Reverse transcriptase (RT)-PCR analysis of the gut-specific knockdown effect of RNAi lines of (B)CG11163 and (C)CG8632. (D) Expression of CG31860 was not detected in the gut. rp49 was used as the loading control. Analysis of CG6672 (dZnT7) and CG5130 (dZnT1h) are described in Figures 4 and 5 respectively. [file 1741-7007-11-101-S2.doc]

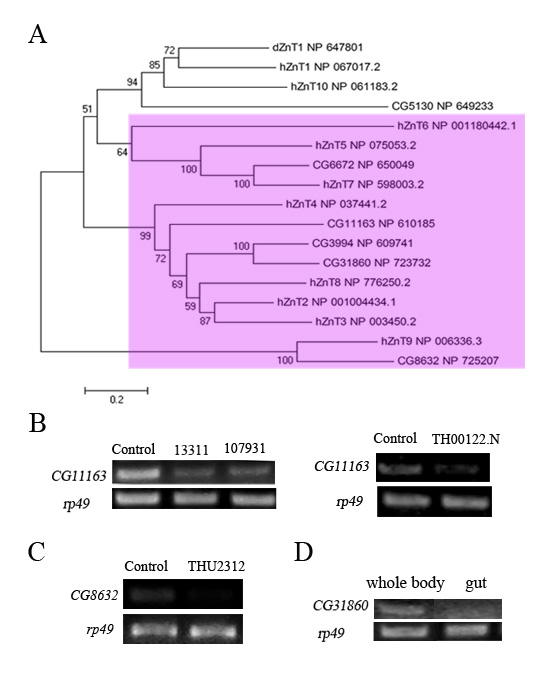


**Figure S2**. Phylogenetic analysis of ZnTs in *Drosophila* (as compared to those in human) and the RNAi effects of some dZnT lines. RNAi lines are from Vienna *Drosophila* RNAi Center (VDRC) or custom made in Tsinghua Fly Center. A) Phylogenetic tree revealing the relationship among human and *Drosophila* ZnT members. The predicted intracellular ZnTs are in pink. All human ZnTs were used respectively as queries to do series of BLASTP searches in the genome of *D. melanogaster*. Tree was generated by using ClustalX version 1.81 and displayed by Treeview. B-D) RT-PCR analysis of gut-specific knockdown effect of RNAi lines of *CG11163* (B), and *CG8632* (C). Expression of *CG31860* was not detected in the gut (D). *rp49* was used as the loading control. Analysis of *CG6672* (*dZnT7*) and *CG5130* (*dZnT1h)* are described in Fig. 4 and 5 respectively.
