# Supplementary material for: Functional studies of Drosophila zinc transporters reveal the mechanism for dietary zinc absorption and regulation
Source: BMC Biol. 2013 Sep 24;11:101. doi: 10.1186/1741-7007-11-101 (PMC4015762; doi:10.1186/1741-7007-11-101)
Supplement: Additional file 3: Table S1 — Analysis of intracellular ZnTs for their roles in dietary zinc absorption in Drosophila. (A) Phenotypes of individual RNA interference (RNAi) lines driven by da-GAL4, a ubiquitous GAL4 line, under normal or zinc-limited conditions. (B) Phenotypic analysis of midgut-specific knockdown of individual RNAi lines on normal or zinc-limited food. NP3084 was used as the midgut GAL4 driver. [file 1741-7007-11-101-S3.doc]

| Ubiquitous RNAi fly normal food 300μM EDTA  (*da*-GAL4) |
| --- |
| *CG11163*-RNAi normal normal  *dZnT7*-RNAi(*CG6672*-RNAi) Lethal at 3^rd^ larvae stage  *CG31860*-RNAi Lethal at embryo or 1^st^ larvae stage  *CG8632*-RNAi Lethal at late pupae stage |

B.

| Gut RNAi fly 100μM EDTA 300μM EDTA  (*NP3084*-GAL4) |
| --- |
| *CG11163*-RNAi normal  *dZnT7*-RNAi(*CG6672*-RNAi) normal  *CG31860*-RNAi normal  *CG8632*-RNAi normal  *dZnT1*-RNAi normal Arrested at 3^rd^ larvae stage  *CG11163*-RNAi *+dZnT1*-RNAi normal Arrested at 3^rd^ larvae stage  *dZnT7*-RNAi *+dZnT1*-RNAi normal Arrested at 3^rd^ larvae stage  *CG31860*-RNAi *+dZnT1*-RNAi normal Arrested at 3^rd^ larvae stage  *CG8632*-RNAi *+dZnT1*-RNAi normal Arrested at 3^rd^ larvae stage |

**Table S1**. Analysis of intracellular ZnTs for their roles in *Drosophila* dietary zinc absorption. A) Phenotypes of individual RNAi lines driven by *da-GAL4*, a ubiquitous GAL4 line, under normal or zinc-limited conditions. B) Phenotypic analysis of midgut-specific knockdown of individual RNAi lines on normal or zinc-limited food. *NP3084* was used as the midgut GAL4 driver.
